# Screening of Quality Markers During the Processing of *Reynoutria multiflora* Based on the UHPLC-Q-Exactive Plus Orbitrap MS/MS Metabolomic Method

**DOI:** 10.3389/fphar.2021.695560

**Published:** 2021-08-11

**Authors:** Junqi Bai, He Su, Youling Liang, Xuhua Shi, Juan Huang, Wen Xu, Jing Zhang, Lu Gong, Zhihai Huang, Xiaohui Qiu

**Affiliations:** ^1^ The Second Clinical Medical College of Guangzhou University of Chinese Medicine, Guangdong Provincial Hospital of Traditional Chinese Medicine, Guangzhou, China; ^2^ Key Laboratory of Quality Evaluation of Chinese Medicine of Guangdong Provincial Medical Products Administration, Guangzhou, China; ^3^ Guangdong Key Laboratory of Chirality Research on Active Components of Traditional Chinese Medicine, Guangzhou, China

**Keywords:** processing, discrimination, metabolomics, UHPLC-Q-Exactive MS, *Reynoutria multiflora* (Thunb.) Moldenke

## Abstract

The root of *Reynoutria multiflora* (Thunb.) Moldenke (syn: *Polygonum multiflorum* Thunb.) is a distinguished herb that has been popularly used in traditional Chinese medicine. The raw *Reynoutria multiflora* (RRM) should be processed by steaming before use, and the processing time is not specified in the processing specification. Our previous studies showed that the efficacy and toxicity of processed *Reynoutria multiflora* (PRM) at different processing times were inconsistent. A comprehensive identification method was established in this study to find a quality marker of raw *Reynoutria multiflora* (RRM) and processed *Reynoutria multiflora* (PRM) with different processing times. Metabolomics based on ultra-high-performance liquid chromatography tandem quadrupole/electrostatic field orbitrap high-resolution mass spectrometry (UHPLC-Q-Exactive plus orbitrap MS/MS) was used in this study. Using the CD.2 software processed database, multivariate statistical analysis methods coupled with cluster analysis and heatmap were implemented to distinguish between RRMs and PRMs with different processing times. The results showed that RRM and PRMs processed for 4, 8, 12, and 18 h cluster into group 1, and PRM processed for 24 and 32 h into group 2, indicating that it can effectively distinguish between the two groups and twenty potential markers, made the highest contributions to the observed chemical differences between two groups. Among them, tetrahydroxystilbene-O-hexoside-O-galloyl and sucrose can be used to identify PRM processed for 24 h. Therefore, the properties of RRM changed after 24 h of processing, and the quality markers were screened to distinguish RRM and PPM. It can also be used as an important control technology for the processing of RM, which has wide application prospects.

## Introduction

*Reynoutria multiflora* (Thunb.) Moldenke (RM) is commonly known as He-shou-wu in China and has been widely used in TCM clinical practice for centuries (Li et al., 2017). Both RRM and PRM are officially listed in the Chinese Pharmacopoeia. RRM can provide detoxification, carbuncle elimination, and bowel relaxation. PRM can tonify the liver and kidneys and is used as a medicinal tonic and hair blacking. According to Chinese medicine theory, RRM should be processed before use by steaming with or without black soybean extract, which significantly reduces toxicity and strengthens tonifying effects (Qiu et al., 2008; Dong et al., 2014). However, in recent years, RM have witnessed an increasing number of reports of adverse reactions, especially liver injury, caused by taking *Reynoutria multiflora* and products prepared from *Reynoutria multiflora*. The United Kingdom has recommended *using Reynoutria multiflora* with caution (But et al., 1996; Gabriela et al., 2004; Kyoung et al., 2011; Banarova et al., 2012; Lei et al., 2015; Lin et al., 2015; Zhang et al., 2019). The liver toxicity of PRM is at odds with the traditional use of PRM for tonifying the liver and kidney.

The processing of traditional Chinese medicines is a unique pharmaceutical technology derived from the theory of traditional Chinese medicine (Cai et al., 2012). This processing has played a prominent role in the clinical practice of traditional Chinese medicine for thousands of years and ensures the safety and effectiveness of treatment. The processing methods involve the treatment using different temperatures, times, solvents, and excipients, during which component content and appearance changed. These changes are closely related to the properties and efficacy of these medicines. Therefore, it is extremely important to study the changes in chemical components during processing.

A previous study showed that RM, which was processed for 24–32 h, could improve the hematopoietic function of blood deficiency rats, whereas RRM could not (Qiu et al., 2008; Zhuo, 2012). Researchers proved that Rats that were given RRM and PRM by gavages for 1 month and 3 months exhibited early cholestasis. The toxicity index of the RRM group was better than that of the PRM group (Chen, 2012). The results showed that the processing of RM reduced toxicity and increased efficiency.

The main chemical components of RM are several secondary metabolites, among which stilbene glycosides, anthraquinone, and polyphenols are the most representative (Choi et al., 2007; Sun et al., 2015). Many studies have shown that the content of the chemical components of RM changes during processing. In the previous study, we also demonstrated that the contents of some chemical compounds are changed during the processing process. The combined anthraquinones are hydrolyzed and the free anthraquinones, like emodin and physcion, are actually increased. The content of stilbene glycosides decreases gradually with processing (Qiu et al., 2006; Chen et al., 2012; Zhang et al., 2016). Some new compounds have been reported, such as 2, 3-dihydro-3, 5-dihydroxy-6-methyl-4 (H)-pyran-4-one (DDMP), and 5-hydroxymethyl-furfural (Liu et al., 2008; Liu, 2009; Liu et al., 2009). These reports showed that processing changed the chemical compositions and the changes at different time points were very complex and required comprehensive and objective analysis.

Metabolomics is the comprehensive analysis of metabolites and probes global chemical differences, enabling the most effective chemical markers to be identified for QC of TCM. Thus, metabolomics combined with chemometrics is increasingly being utilized for the QC of TCM. Therefore, in the present study, a metabolomic analysis based on an effective and sensitive ultra-performance liquid chromatography tandem quadrupole/electrostatic field orbitrap high-resolution mass spectrometry (UHPLC-Q-Exactive plus orbitrap MS/MS) method was established to distinguish between RRM and PRM with different processing times. Multivariate statistical methods, including PCA and OPLS-DA, were applied to identify chemical markers that can be used to distinguish between RRM and PRM with various processing times. A heatmap was used to identify trends for these markers. This study is a feasible strategy for the control of RM processing technology and other quality-influencing factors.

## Materials and Methods

### Materials

RRM and PRMs that had been processed for 4, 8, 12, 18, 24, and 32 h samples were provided by Shanghai Dehua National Pharmaceutical Products CO., Ltd, and the corresponding batch numbers were HSW2018051101-S, HSW2018051101-4 H, HSW2018051101-8 H, HSW2018051101-12 H, HSW2018051101-18 H, HSW2018051101-24 H, and HSW2018051101-32 H. They were authenticated by Professor Zhihai Huang and deposited in the Materials Medica Preparation Lab of the Second Affiliated Hospital of the Guangzhou University of Chinese Medicine.

Trans-2, 3, 5, 4′-tetrahydroxystilbene-2-O-β-D-glucopyranoside (THSG) was obtained from Yuanye Bio-Technology Co., Ltd. (No. B20185, P27A11P107214, purity ≥98%, Shanghai China) was used in this study. Acetonitrile (HPLC grade) and methanol (HPLC grade) were supplied by E. Merck (Darmstadt, Germany), formic acid (HPLC grade) was purchased from Fisher (United States), and ultrapure water was prepared by a Milli-Q water purification system (Millipore, MA, United States).

### Preparation of Samples

All the samples were prepared using the following method: 1 g sample powder was ultrasonicated for 30 min with 25 ml of 70% ethanol, followed by filtration and then evaporated the filtrate. 5 ml of ultrapure water was added to dissolve the residue and then extracted twice with 15 ml of ethyl acetate. The resulting mixture was combined with an ethyl acetate solution and evaporated over a water bath; after that, 1 ml of methanol was added to dissolve the residue and centrifugation (15,000 rpm, 4°C) for 10 min by a 1.5 ml centrifuge tube. Finally, the supernatant of the treated samples was injected into the UPLC-Q-Exactive plus orbitrap MS/MS system.

### UPLC-Q-Exactive Plus Orbitrap MS/MS Analysis

#### Liquid Chromatography

All the samples were analyzed using an Ultimate 3000 UPLC system (Dionex, United States) that was controlled with Thermo Xcalibur software (Thermo Fisher Scientific, United States). The samples were separated using a Kinetex UPLC C18 column (1,002.1 mm, 1.7 µm) (Phenomenex, United States). The mobile phase consisted of solvent A (0.1% formic acid) and solvent B (acetonitrile). Gradient elution was applied using the following optimized gradient program: 8–8% B at 0–3 min, 8–28% B at 3–25 min, 28–40% B at 25–26 min, 40–50% B at 26–28 min, 50–70% B at 28–30 min, 70–90% B at 30–32 min, and 90–90% B at 32–35 min. The flow rate was kept at 0.4 ml/min, the sample injection volume was 1 μL, and the column temperature was maintained at 25°C.

### Mass Spectrometry

Mass spectrometry was performed on a Q-Exactive Plus™ quadrupole-Orbitrap mass spectrometer (Thermo Fisher Scientific, United States) in negative ion mode. The scan mass range was set at m/z 100–1,200. The parameter settings were as follows: a full scan and fragment spectral resolution of 70,000 FWHM and 17, 50 FWHM, respectively; the capillary temperature was 350°C; auxiliary gas heater temperature was 350°C; spray voltage was −3.2 KV; sheath gas flow rate was 40 Arb; auxiliary gas flow rate was 15 Arb; S-lens RF level was set at 50. The acquisition mode of stepped NCE (normalized collision energy) was used with settings of 30, 50, and 70 eV. The accumulated resultant fragment ions were injected into the Orbitrap mass analyzer for single-scan detection.

Considering the possible elemental composition of the RM components, the types and quantities of expected atoms were set as follows: carbon ≤50, hydrogen ≤200, oxygen ≤20, nitrogen ≤3. The accuracy error threshold was fixed at 5 ppm.

### Data Processing

The samples were collected and analyzed by UPLC-Q-Exactive plus orbitrap MS/MS. The original data files were imported into Compound Discoverer 2.0 software (Thermo Fisher, United States). The retention time RT = 0.2 min, mass = 10 ppm, and other parameters were selected as the default values for peak extraction and peak alignment. The resulting data matrix was imported into Simca-p (version 14.1) to perform a multivariate statistical analysis. This analysis consisted mainly of a principal component analysis (PCA) and an orthogonal partial least squares discriminant analysis (OPLS-DA). Unsupervised PCA was used to perform a preliminary correlation analysis of the data matrices. Then, the differences in the metabolites in RRM and PRMs with different time processing times were detected using OPLS-DA. The corresponding VIP values were calculated using the OPLS-DA model. The VIP value represents the difference between the considered variables. AVIP value above 1.5 indicated components that play an important role in differentiating between RRM and PRM. The significance of differences was tested by using SPSS 22.0 (IBM SPSS Statistics, United States) to perform a nonparametric test on two independent samples, where *p* < 0.05 was considered significant. Only components with VIP >1.5 and *p* < 0.05 were selected as potential markers. A heatmap was plotted to visualize the variations in potential markers for separating samples with processing times into different groups.

## Results and Discussion

### Identification of Appearance

**FIGURE 1:**
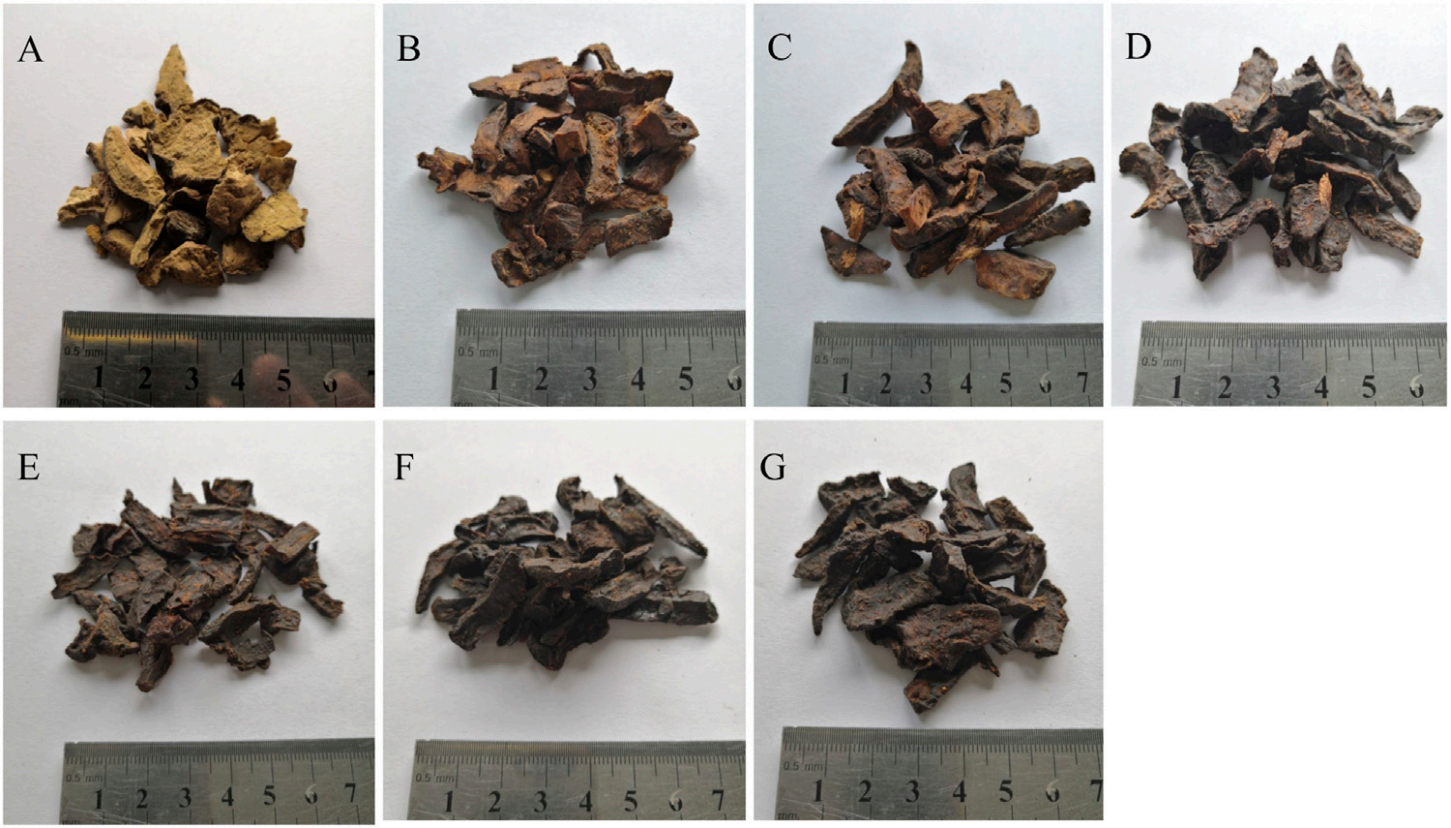
Characters of raw *Reynoutria multiflora* (RRM) and processed *Reynoutria multiflora* (PRM). (**(A)**: RRM, **(B)**: PRM 4 h, **(C)**: PRM 8 h, **(D)**: PRM 12 h, **(E)**: PRM 18 h, **(F)**: PRM 24 h, **(G)**: PRM 32 h). The appearance, section, and texture all change regularly.

The appearance of the samples was first observed in Figure 1: RRM was yellow-brown and turned to tan color after 4 and 8 h of processing, mainly black-brown after 12 and 18 h of processing and black-brown color after 24 and 32 h of processing. RRM was crisp and powdery, and the texture became harder as the processing time increases. That is, RRM was no longer powdery after 4 h of processing. The samples were sliced without keratinization after 4 and 8 h of processing. After processing for 12 and 18 h, the sample slices were incompletely keratinized, and the center was mostly brown without cutinization. After 24 and 32 h of processing, the sample slices were completely keratinized with a metallic luster. Based on the changes in appearance, the important processing points were identified as 4 and 24 h. After 4 h of processing, the samples were no longer powdery, and the RM slices began to turn cutinize brown, and the color continued to deepen up as the extension of processing time. After 24 h of processing, the samples began to cutinize completely and exhibited a metallic luster, and the color transitioned to black-brown.

### Comparative Chemical Profiling Analysis of RRM and PRM with Different Processing Times

**FIGURE 2:**
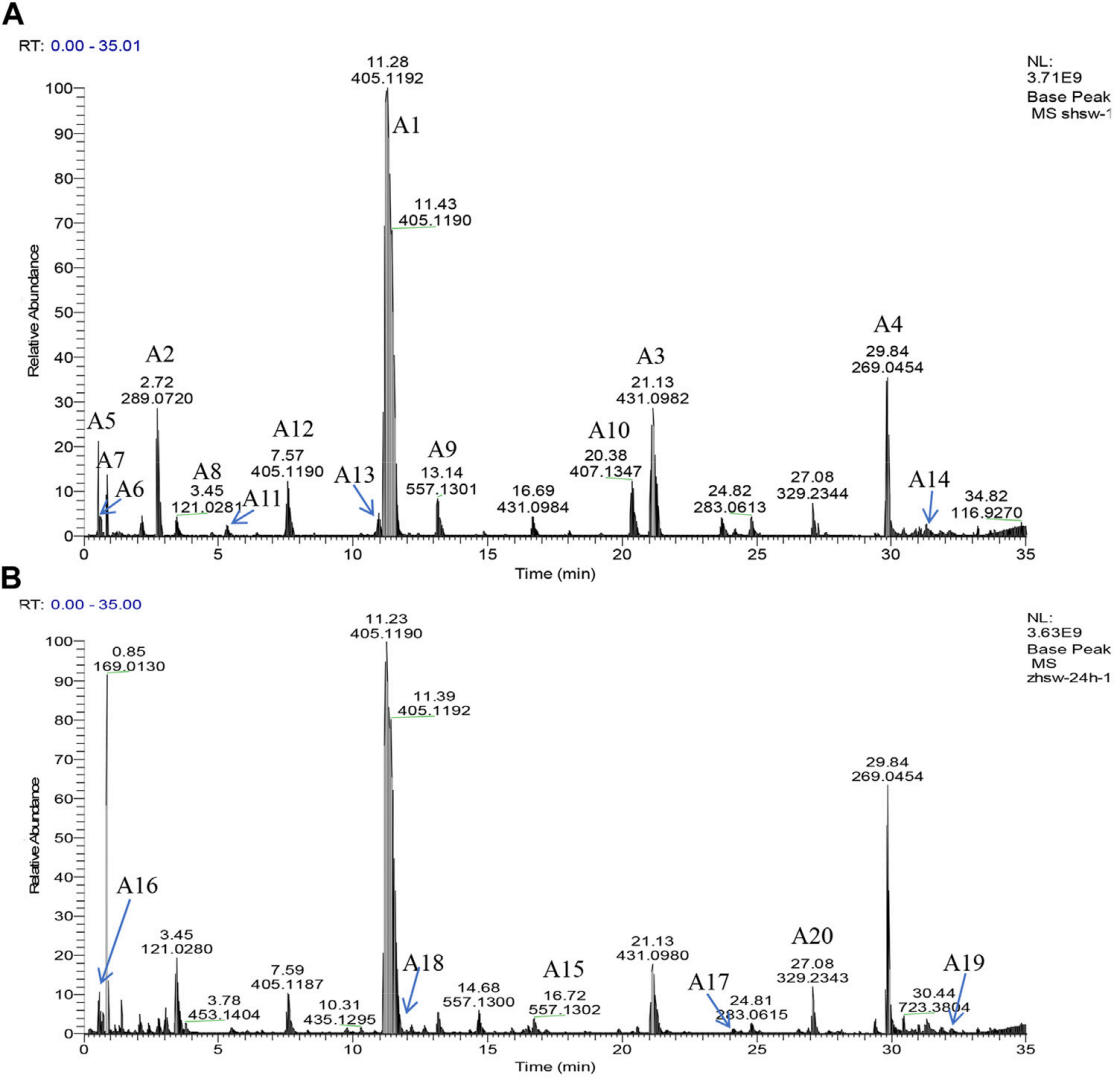
Representative base peak intensity chromatograms of samples of *Reynoutria multiflora* with different processing times derived from UHPLC-Q Exactive plus orbitrap MS/MS. **(A)** Raw *Reynoutria multiflora* (RRM), **(B)** Processed *Reynoutria multiflora* (PRM 24 h). Refer to Table 1 for compound labels in the figure.

In this study, chemical profiling of RRM and PRM with different processing times was performed by UHPLC-Q-Exactive plus orbitrap MS/MS, where the base peak ion chromatograms of the samples are shown in Figure 2. The PRM samples with different processing times exhibited similar contours, but there were considerable differences in the details; for example, the peak for catechin at 2.72 min was not observed in the PRM sample processed for 24 h.

### Discrimination of RRM and PRM With Different Processing Times Based on Metabolomics

Multivariate statistical methods, including PCA and OPLS-DA, were employed to discriminate RRMs and PRMs with different processing times and find potential classification markers.

### Multivariate Statistical Analysis

#### Principal Component Analysis of RRM and PRM Samples

**FIGURE 3:**
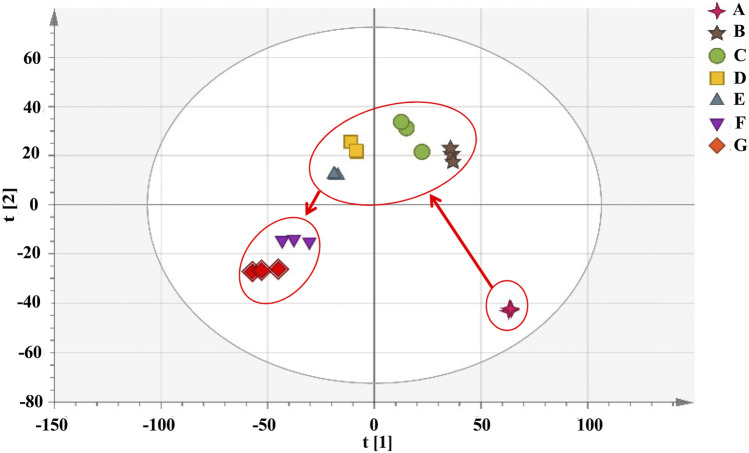
The score plots of PCA model for samples of *Reynoutria multiflora* with different processing times analysis. R2X and Q2 values of the PCA model are 0.953 and 0.871, respectively. **(A)**: raw *Reynoutria multiflora* (RRM), **(B)**: processed *Reynoutria multiflora* (PRM) 4 h, **(C)**: PRM 8 h, **(D)**: PRM 12 h, **(E)**: PRM 18 h, **(F)**: PRM 24 h, **(G)**: PRM 32 h.

PCA was performed on the UHPLC-Q-Exactive plus orbitrap MS/MS data for samples of RRM and PRM with different processing times that have been subjected to several pretreatment procedures to identify grouping trends. The PCA score plot is shown in Figure 3. The samples can be clearly classified into three groups: RRM, PRM processed for 4–18 h, and PRM processed for 24–32 h. The PCA results indicated that the metabolites changed significantly after being processed for 4 and 24 h; the samples that were processed for 4, 8, 12, and 18 h and the samples that were processed for 24 and 32 h were mixed together, indicating that the change of metabolites is significant in these two time intervals.

#### Cluster Analysis of RRM and PRM Samples

**FIGURE 4:**
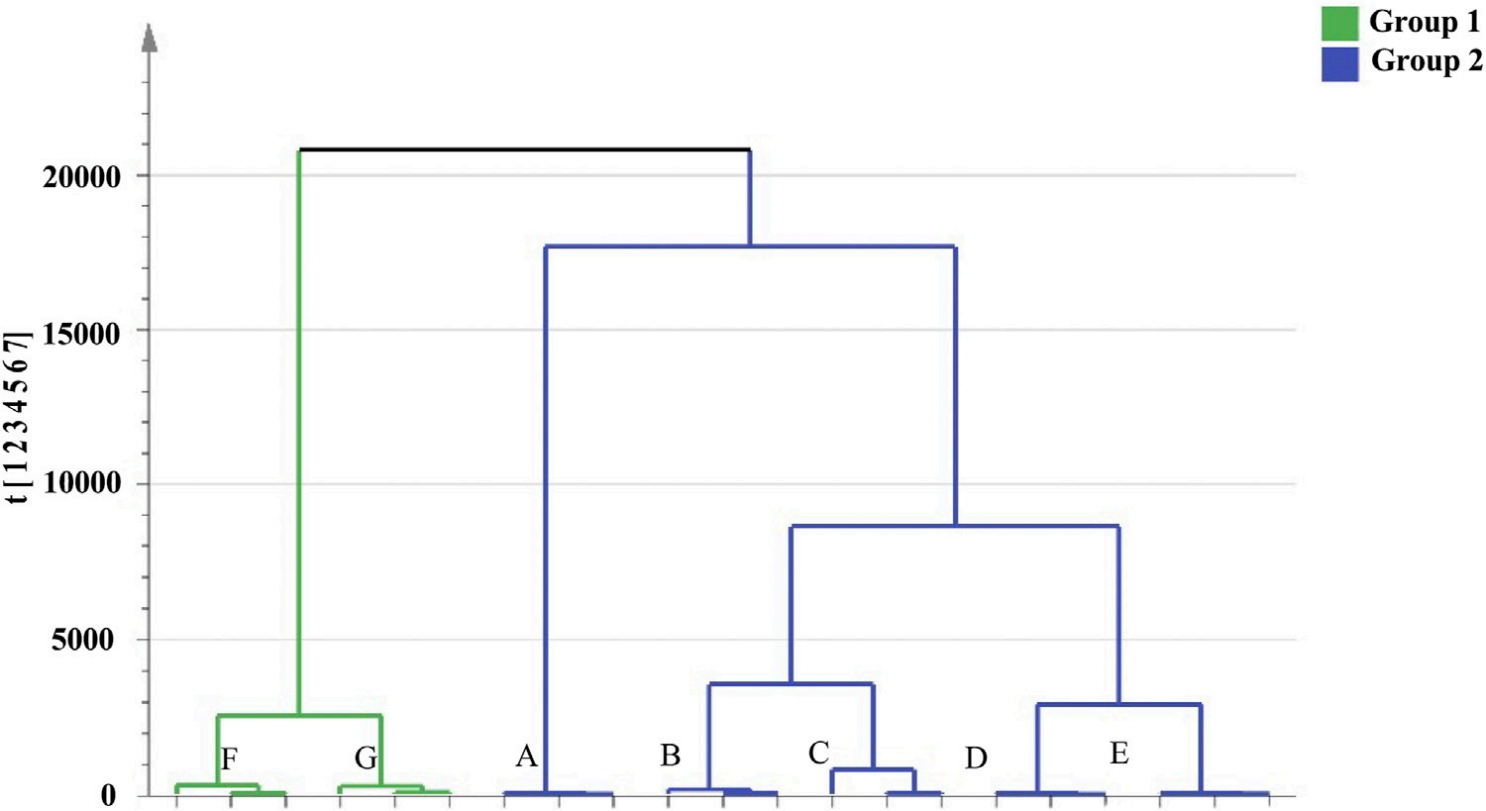
Dendrograms of hierarchical cluster analysis (HCA) for samples of raw *Reynoutria multiflora* (RRM) and processed *Reynoutria multiflora* (PRM). (Group1: **(F)**: PRM 24 h, **(G)**: PRM 32 h; Group 2: **(A)**: RRM, **(B)**: PRM 4 h, **(C)**: PRM 8 h, **(D)**: PRM 12 h, **(E)**: PRM 18 h).

Cluster analysis showed that all the samples were divided into two categories (Figure 4). RRM and PRM processed for 4, 8, 12, and 18 h were clustered into a group, indicating that although the chemical composition changed significantly during the processing time of 4–18 h, the corresponding PRM samples were still clustered with RRM, that is, PRM processed for 4–18 h retained the properties of RRM. The PRM samples processed for 24 and 32 h were grouped together and divided with other samples, indicating that the RRM properties were extremely changed by processing for 24 and 32 h, which was consistent with the results of using the blood deficiency animal model, which was used to determine the best processing technology.

#### Discriminant Analysis of RRM and PRMs Using the Orthogonal Partial Least Squares Method

**FIGURE 5:**
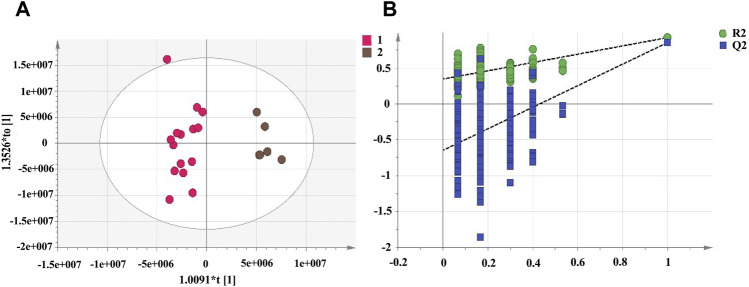
The score plots of OPLS-DA model for raw *Reynoutria multiflora* (RRM) and processed *Reynoutria multiflora* (PRM) analysis. R2Y and Q2 values of the OPLS-DA model are 0.925, 0.856, respectively. (**(A)**: Left: RRM, PRM for 4, 8, 12, and 18 h; right: PRM for 24 and 32 h.) **(B)**: cross-validation plot of OPLS-DA model with 200 permutation tests.

PCA cannot assign the class membership of unknown test samples. OPLS-DA performs grouping and determines differences between grouped samples and has a higher classification and prediction capacity than PCA. The results of the PCA and cluster analysis were used to classify the UHPLC-Q-Exactive plus orbitrap MS/MS data of RRM and PRMs processed for 4, 8, 12, and 18 h as group 1 and PRMs processed for 24 and 32 h as group 2, which were further analysed by OPLS-DA. The OPLS-DA score plots (Figure 5) show that the metabolic profiles of group 1 and group 2 could be completely separated, suggesting significant differences between the samples in these two groups. Two hundred rounds of random permutations were performed to verify the established OPLS-DA model, and the results indicated that the model was reliable (R2Y = 0.925, Q2 = 0.856).

To select chemical markers, the ions were further screened based on the VIP values. The higher the ions VIP value was, the further the VIP was from the origin. The variables with VIPs >1.5 and *p* < 0.05 in the nonparametric test were considered as potential chemical markers for distinguishing the two groups. Combining the results of the identification analysis and removing repetitive, aglycone, and isotope effects yielded, a total of 20 potential chemical marker metabolites were identified, which was shown in Table 1.

**TABLE 1:** Details of potential chemical markers.

No	t_R_ (min)	[M-H]^-^ (*m/z*)	Formula	Fragment ions (*m/z*)	Error (ppm)	Identification	VIP	*p*
A1	11.25	405.1190	C_20_H_21_O_9_	243.0653	−0.260	Trans-THSG^a^	40.27	0.00
A2	2.72	289.0720	C_15_H_13_O_6_	289.0712 245.0811 205.0492 203.0699 179.0335 165.0178 151.0386 125.0227 109.0278	0.825	Catechin^a^	14.01	0.00
A3	21.13	431.0982	C_21_H_19_O_10_	431.0982 269.0454	−0.394	Emodin-8-O-glucoside^a^	10.50	0.00
A4	29.84	269.0454	C_15_H_9_O_5_	269.0453	−0.545	Emodin^a^	10.06	0.00
A5	0.53	341.1096	C_12_H_21_O_11_	179.0546 161.0440 143.0333 119.0334 101.0228 89.0228 71.0122 59.0123	1.951	Sucrose Wang et al. (2016)	8.83	0.00
A6	0.84	123.0073	C_6_H_3_O_3_	123.0071		2,5-Furandicarboxaldehyde Zhang (2018)	7.01	0.00
A7	0.85	169.0131	C_7_H_5_O_5_	169.0128 125.0228	−6.784	Gallic acid^a^	6.93	0.00
A8	3.44	121.0281	C_7_H_5_O_2_	121.0278	−11.590	Benzoic acid^a^	5.18	0.00
A9	13.14	557.1302	C_27_H_25_O_13_	405.1178 313.0555 243.0654 169.0127	0.244	Tetrahydroxystilbene-O-hexoside-O-galloyl Qiu et al. (2013)	5.08	0.00
A10	20.37	407.1345	C_20_H_23_O_9_	245.0812	−0.628	Torachrysone-O-glucoside^a^	4.58	0.00
A11	5.21	289.0721	C_15_H_13_O_6_	289.0712 245.0811 205.0492 203.0699 179.0335 165.0178 151.0386 125.0227 109.0278	1.171	Epicatechin Wang et al. (2016)	4.15	0.00
A12	7.60	405.1191	C_20_H_21_O_9_	243.0653	−0.013	Cis-THSG^a^	4.05	0.00
A13	11.00	441.0824	C_22_H_17_O_10_	289.0710 245.0813 169.0127 125.0227	−0.725	Gallocatechin gallate^a^	2.77	0.00
A14	31.52	325.1841	-	-	-	Unknown (blank interference)	2.31	0.00
A15	16.74	557.1302	C_27_H_25_O_13_	405.1178 313.0555 243.0654 169.0127	0.244	Tetrahydroxystilbene-O-hexoside-O-galloyl Qiu et al. (2013)	2.20	0.00
A16	0.55	439.0854 (M + H_2_SO_4_−H)	C_12_H_21_O_11_	-	-	Sucrose^a^	1.97	0.00
A17	24.12	285.0402	C_15_H_9_O_6_	285.0402	−0.952	Citreorosein^a^	1.90	0.00
A18	11.9	441.0822	C_22_H_17_O_10_	289.0710 245.0813 169.0127 125.0227	−1.179	Epigallocatechin gallate Huang et al. (2018)	1.76	0.00
A19	32.32	339.1995	-	-	-	Unkown (blank interference)	1.57	0.00
A20	27.08	329.2343	C_18_H_33_O_5_	329.2324 229.1434 211.1327 171.1012	2.893	Trihydroxyoctadecamonoenoic acid Galliard et al. (1975)	1.53	0.00

a Compared with standard compounds.

### Heatmap Analysis of RMP and PRMs With Different Processing Times

**FIGURE 6:**
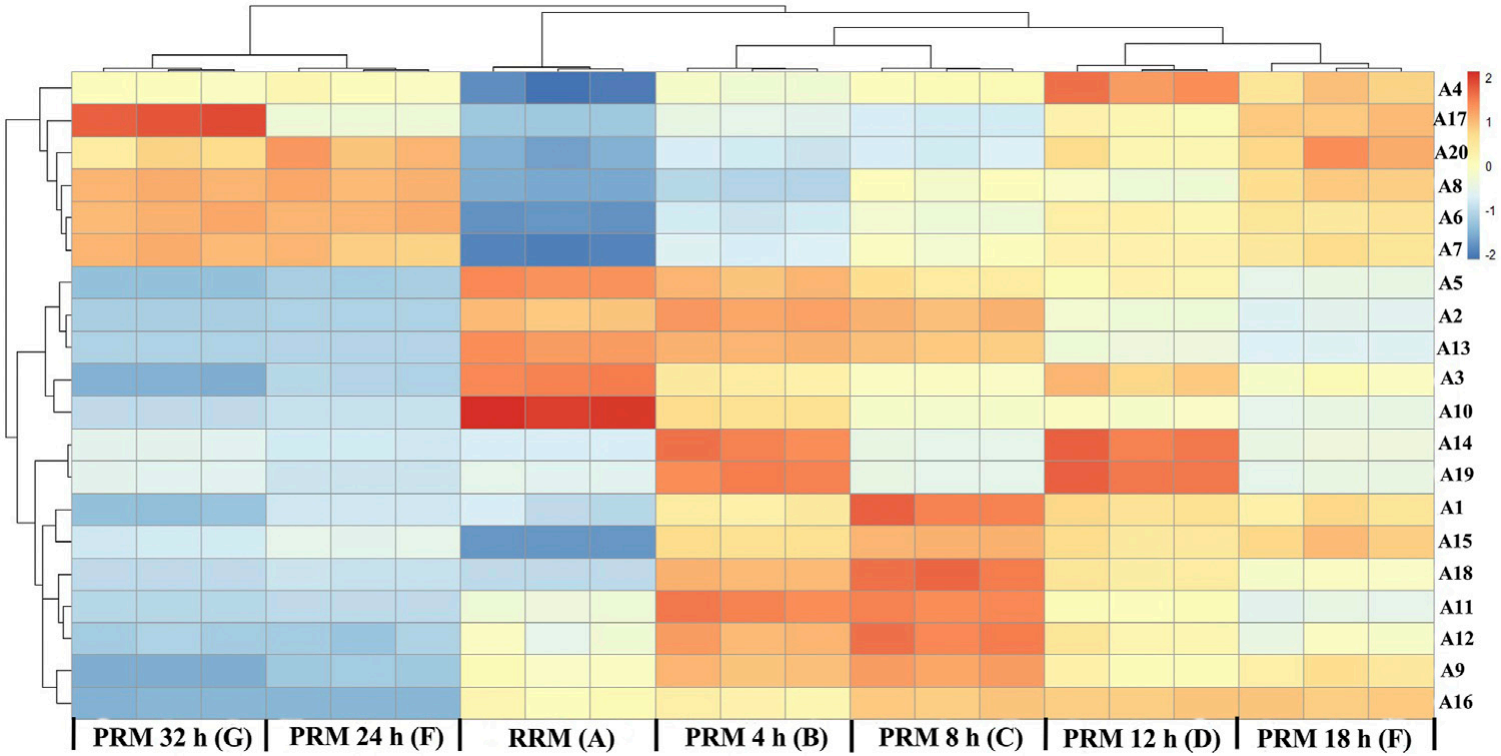
Heat map of potential markers. Yellow in gradient presented the increases. Blue in gradient presented the decreases. (X-axis: **(A)**: raw *Reynoutria multiflora* (RRM), **(B)**: processed *Reynoutria multiflora* (PRM) 4 h, **(C)**: PRM 8 h, **(D)**: PRM 12 h, **(E)**: PRM 18 h, **(F)**: PRM 24 h, **(G)**: PRM 32 h; Y-axis: potential markers, **A4**, **A17**, **A20**, **A8**, **A6**, **A7**, **A5**, **A2**, **A13**, **A3**, **A10**, **A14**, **A19**, **A1**, **A15**, **A18**, **A11**, **A12**, **A9**, **A16**).

The 20 potential markers for RRM and PRMs processed for 4, 8, 12, 18, 24, and 32 h are shown in the heatmap in Figure 6. All the markers were also clearly divided into two main clusters, which are consistent with the results of cluster analysis and PCA. Colors indicate the signal intensity of each metabolite: the red boxes indicate that the metabolite content was higher than the mean level of the respective sample and the blue boxes indicate low metabolite content. Obviously, six metabolites (A4 (emodin), A6 (2, 5-furandicarboxaldehyde), A7 (gallic acid), A8 (benzoic acid), A17 (citreorosein), A20 (trihydroxyoctadecamonoenoic acid)) showed a higher level of group 2 whereas other 14 metabolites (A1 (trans-2, 3, 5, 4′-tetrahydroxystilbene-2-O-β-D-glucopyranoside, trans-THSG, A2 (catechin), A3 (emodin-8-O-glucoside), A5 (sucrose), A9 (tetrahydroxystilbene-O-hexoside-O-galloyl), A10 (torachrysone-O-glucoside), A11 (epicatechin), A12 (cis-THSG), A13 (gallocatechin gallate), A14 (unknown), A15 (tetrahydroxystilbene-O-hexoside-O-galloyl), A16 (sucrose), A18 (epigallocatechin gallate), A19 (unknown)) showed the opposite.

Detailed analysis showed that the contents of A6, A7, and A8 gradually increased with the increase of processing time, which can be used as quality markers to distinguish RRM and PRM. The content of compound 20 increased regularly before processing for 18 h and remained stable until 32 h. The content of compound 17 was the highest in the samples processed for 24 h, which gradually increased before but decreased significantly at 32 h. The change characteristics of compound A4 are similar to compound A17, but the content of A4 is the highest in the sample processed for 12 h, then it gradually decreased to the content of 8 h in processed products.

The contents of A2, A3, A5, A10, and A13 gradually decreased with the increase of processing time, which also can be used as quality markers to distinguish RRM and PRM. Compounds 14 and 19 could not be used as markers to distinguish RRM and PRM. The contents are higher only in the samples processed for 4 and 12 h but lower in other samples. The content of compounds A1, A11, A12, A15, and A18 was low level in RRM, reached the maximum after processing for 8 h, and then gradually decreased to the same level as RRM. Compounds A9 and A16 were very interesting. Their content in RRM was clearly defined. The content of compound A9 was the highest after processing for 8 h, slightly decreased after processing for 18 h, and significantly decreased to a level lower than RRM after processing for 24 h. The content of compound A16 gradually increased by processing for 4–18 h but decreased significantly to a level much lower than RRM after processing for 24 h.

Consequently, compounds A2, A3, A5, A6, A7, A8 A9, A10, A13, and A16 can be used to identify RRM and PRM, and tetrahydroxystilbene-O-hexoside-O-galloyl (A9) and sucrose (M + H_2_SO_4_) (A16) are more likely to be used as quality markers for RRM and PRM because their contents significantly decrease processing for 24 h, far lower than those in RRM and PRM for 4–18 h. RM is a traditional Chinese medicine produced all over China. In view of the large changes in the chemical composition of RM, it is impossible to adjust the components changes with the processing time unless the content of compounds changes in a constant proportion with the processing time.

## Conclusion

In this study, a UHPLC-Q-Exactive plus orbitrap MS/MS method was established to distinguish samples of RRM and PRM with different processing times. The PRM appearance changes in a well-defined manner with the processing time. 24 h of processing resulted in cutinization and a metallic luster, and both the inside and outside of the sample become dark brown. A multivariate statistical analysis coupled with cluster analysis and heatmap generation showed that RRM and PRMs with a processing time of 4, 8, 12, and 18 h were clustered together, while PRMs with a processing time of 24 and 32 h were clustered into another group, where the samples in each group have similar appearances. The 20 potential markers made the highest contributions to the observed chemical differences between RRM and PRMs. Through the comparative analysis of heatmap, ten quality markers were screened out, of which 8 markers showed regular changes, 3 markers increased regularly, 5 markers decreased gradually, and the other two markers (tetrahydroxystilbene-O-hexoside-O-galloyl and sucrose (M + H_2_SO_4_)) can be used to identify samples processing for 24 h. Further chemical analysis is needed to correlate the clear changes in the contents of the marker compounds with the processing time and the exact limit of the changes after processing for 24 h should be determined RM. The proposed method can be used as an important control technology for traditional Chinese medicine processing and has wide application prospects.

## Data Availability

The raw data supporting the conclusions of this article will be made available by the authors without undue reservation.
