# Supplementary material for: Screening of Quality Markers During the Processing of Reynoutria multiflora Based on the UHPLC-Q-Exactive Plus Orbitrap MS/MS Metabolomic Method
Source: Front Pharmacol. 2021 Aug 11;12:695560. doi: 10.3389/fphar.2021.695560 (PMC8385779; doi:10.3389/fphar.2021.695560)
Supplement: Supplementary file 1 [file DataSheet1.docx]

Appendix

1. Standard chromatogram (total ion flow chromatogram/base peak)

1. Trans-THSG and cis-THSG standard chromatogram (t_R_ =11.45 min trans-THSG, t_R_ =7.51 min cis-THSG

1. Catechin standard chromatogram

1. emodin-8-O-glucoside standard chromatogram
2. emodin standard chromatogram

1. 2,5-furandicarboxaldehyde standard chromatogram

1. gallic acid standard chromatogram

1. benzoic acid standard chromatogram

1. torachrysone-O-glucoside standard chromatogram

1. gallocatechin gallate standard chromatogram

1. citreorosein standard chromatogram
